# The Importance of Blood Is Infinite: Conceptions of Blood as Life Force, Rumours and Fear of Trial Participation in a Fulani Village in Rural Gambia

**DOI:** 10.1371/journal.pone.0160464

**Published:** 2016-08-15

**Authors:** Sarah O’Neill, Susan Dierickx, Joseph Okebe, Edgard Dabira, Charlotte Gryseels, Umberto d’Alessandro, Koen Peeters Grietens

**Affiliations:** 1 Unit of Medical Anthropology, Department of Public Health, Institute of Tropical Medicine, Antwerp, Belgium; 2 Amsterdam Institute of Social Science Research, University of Amsterdam, Amsterdam, The Netherlands; 3 RHEA, Centre of Expertise on Gender, Diversity and Intersectionality, Vrije Universiteit Brussels, Brussels, Belgium; 4 Medical Research Council Gambia, Fajara, The Gambia; 5 University of Antwerp, Antwerp, Belgium; 6 London School of Hygiene and Tropical Medicine, London, United Kingdom; 7 School of Tropical Medicine and Global Health, Nagasaki University, Nagasaki, Japan; 8 Partners for Applied Social Sciences (PASS) International, Tessenderlo, Belgium; University of California Los Angeles, UNITED STATES

## Abstract

**Background:**

Clinical trials require high levels of participation and low drop-out rates to be successful. However, collecting blood samples from individuals recruited into clinical trials can be challenging when there is reticence about blood-taking. In addition to concerns regarding the feasibility of medical research, fears of ‘blood-stealing’ and ‘blood-selling’ have ethical implications related to cultural sensitivity and informed consent. This study explores anxieties around blood-taking during a malaria treatment trial in the Gambia.

**Methods:**

This case study is based on ethnographic research in one theoretically selected village due to the high reticence to screening for the clinical trial ‘*Primaquine's gametocytocidal efficacy in malaria asymptomatic carriers treated with dihydroartemisinin-piperaquine’* carried out in the Gambia between 2013 and 2014. Data collection tools included in-depth interviews, participant observation, informal conversations and group discussions.

**Results:**

In total only 176 of 411 habitants (42%) in the village accepted having a bloodspot taken to screen for malaria. Although trial recruitment was initially high in the village, some families refused screening when rumours started spreading that the trial team was taking too much blood. Concerns about ‘loss of blood’ were equated to loss of strength and lack of good food to replenish bodily forces. Families in the study village were concerned about the weakness of their body while they had to harvest their crops at the time of recruitment for the trial.

**Conclusion:**

A common recommendation to prevent and avoid rumours against public health interventions and trials is the provision of full and consistent information during the consent procedure, which is assumed to lead to more accurate knowledge of the purpose of the intervention and increased trial participation. However, even when information provision is continuous, the emergence of rumours can be related to times of uncertainty and perceptions of vulnerability, which are often a reflection of structural inequalities and diverging value orientations between communities and public health institutions.

## Introduction

### Background

Medical research involving human subjects has increased substantially over the last decades [1,2]. Although the number of clinical trials conducted in Sub Saharan Africa is lower than in Europe and the United States, their number has grown significantly, a consequence of the higher availability of resources for public health-orientated research and the need to address the high burden of diseases in these countries [1,2].

Clinical research is regulated by internationally agreed ethical principles, formulated into guidelines, national laws and regulations [3–5]. Nevertheless, the ethical conduct of research specific to developing countries has been subject to discussion [6–8]. People’s decision to participate in medical research and clinical trials in Sub Saharan Africa can be influenced by the perceived benefits [7] and the level of trust patients place on investigators [1,9]. Furthermore, potential trial participants’ understanding of research methods, such as blinding and randomization, and perceptions of risk can influence the decision to participate [1]. Even when procedures are guided by approved protocols and followed by the book, collecting blood or other bodily samples from individuals recruited into clinical trials can be challenging when rumours arise [10–13]. Fears of blood-stealing, body parts trade, sterilization campaigns and the deliberate spreading of diseases in relation to public health interventions have been documented since colonial times [10,13–15], and continue to influence medical research and clinical trials [6,12].

When rumours arise, investigators often respond by intensifying community sensitization and providing additional information. At times, research participants and influential members of the community are taken to the clinic and to the laboratories to demonstrate scientific procedures. Such measures are often facilitated by fieldworkers who speak the local languages and are able to explain the scientific procedures. Some trials strongly encourage the placement of fieldworkers in the study villages and thus fieldworkers often reside in trial participants’ homes [16]. This seems to have a positive effect on trial participation since misunderstandings and emerging rumours can be dealt with immediately. However, this can also have ethical implications when villagers agree to participate on the basis of friendship, social kinship and trust, rather than with the intention to contribute to the advancement of science and medical research [16–18].

Clinical trials require maximum participation and low drop-out rates to be successful [6,19,20]. An improved understanding of potential study participants’ concerns and beliefs about blood-taking in medical research could improve free and informed decision-making for potential participants and enhance the overall trust relationship between medical institutions and research participants [1]. This case study explores anxieties around blood-taking as a reason for reticence towards screening for the inclusion in a malaria treatment trial in one village in rural Gambia.

## Methods

### Study design

The presented research is a case study. Case studies can be defined as ‘an empirical inquiry that investigates a contemporary phenomenon within its real-life context; when the boundaries between phenomenon and context are not clearly evident; and in which multiple sources of evidence are used’ [21]. It allows for an in-depth understanding of ‘how’ and ‘why’ rumours about blood-sampling emerged, and takes into account the local context [22,23].

This case study used an ethnographic approach. Qualitative data were gathered by triangulating in-depth interviews, focus-group discussions, and participant observation, including informal talks during fieldwork, in order to enhance the reliability and validity of emerging results. Sampling was theoretical and analysis was a process concurrent with data collection in line with a grounded theory approach[24].

The case study was ancillary to the trial ‘*Primaquine's gametocytocidal efficacy in malaria asymptomatic carriers treated with dihydroartemisinin-piperaquine’* (ClinicalTrials.gov: NCT01838902) (hereafter, *Prinogam*), evaluating the effect of different doses of primaquine to clear gametocytes. This health facility based trial was carried out in the Upper River Region and the Central River Region during the 2013 and 2014 rainy seasons [25]. The initial pre-screening was done to determine infection (rapid antigen test kit) and to establish a baseline parasitaemia (microscopy). The pre-screening took place in 56 villages.

For the anthropological case study, a Fula village was theoretically selected from 12 villages in which ethnographic fieldwork during the *Prinogam* trial took place. The selection was based on the substantial reticence in the village, in comparison to other villages participating in the *Prinogam* trial. The study took place in November and December 2013 and in August 2014. Ethnographic research methods were employed to understand reasons for low participation.

### Study site and population

The selected village for the case study comprises 12 compounds located about an hour’s drive south east of Basse, where the Medical Research Council Gambia (MRCG) field station is located. It is two kilometres away from the border with Senegal. According to the census data of 2013, 411 individuals were officially registered as residents, some of whom were absent at the time of screening (see Table 1). All inhabitants were Fulani and lived off cattle herding and subsistence farming (predominantly millet, maize, groundnuts and rice) as well as remittance from family members living in urban areas or from relatives living abroad. There was no electricity in the village and inhabitants fetched water from a nearby well. For healthcare and business, participants either went to Madina Samako, a large village nearby, or to Velingara in Senegal. Basse was perceived to be less accessible and transport less affordable.

**Table 1:** Socio-demographic characteristics of participants (N = 30).

	Men	Women
	n	n
**Occupation**		
Elderly (retired)	3	3
Alkalo (village chief)	1	0
Policeman	1	0
Marabout (traditional healer)	1	0
Imam	1	0
Herder	5	0
Farmer/housework	3	10
Traditional birth attendant	0	2
**Total**	15	15

The principal malaria parasite in The Gambia is *Plasmodium falciparum*, which is transmitted by three mosquito species of the *Anopheles gambiae* complex [26]. Malaria transmission is moderate and highly seasonal, starting during the short rainy season (June-September) and peaking from November until December/January. Malaria prevalence is heterogeneous, with the eastern part of the country having the highest prevalence [27].

Research on treatment-seeking-behaviour for malaria in the Gambia found that in 2003, 63% of respondents took their children to the health centre for malaria treatment and 13% to a health village post [28]. Wiseman et al. (2008) found that 48.6% of respondents went to the hospital for malaria treatment, 21.7% to the health centre and 4.5% to the clinic [29]. Recent evidence suggests that treatment seeking depends on the symptoms, perceptions of the cause and severity as well as the development of the malaria episode, the healing process or the lack of recovery. Patients do not necessarily go to the health centre if they interpret the symptoms of the illness as a self-limiting febrile illness or a folk illness (*Jontinooje/Kajeje*)[30]. A visit to the health centre is often delayed until the patient is seriously sick [30].

### The Prinogam trial

The case study was part of the *Prinogam* trial in which the following procedures were followed:

#### Community sensitization

In agreement with the village chiefs and the village councils, sensitization meetings were held before any research activity started. All inhabitants of the village were invited. At these meetings the purpose of the study and the procedure were explained in detail by the study team, consisting of fieldworkers and nurses from the MRCG.

#### Screening

The *Prinogam* study team moved from compound to compound and verbal consent was taken for a rapid clinical assessment and a Rapid Diagnostic Test (RDT) (pre-screening). Blood from a single finger prick from all non-febrile people was used to prepare a blood slide and perform an RDT. If the RDT was negative, the blood slide was discarded and the person was told that they did not have malaria. Otherwise, the blood slide was taken to the laboratory, stained and read by microscopy to determine the parasite density. Participants with a *Plasmodium falciparum* mono-infection with a density of at least 20 parasites/μL were informed of the result and invited to be taken to the clinic with the MRCG car the following day. After obtaining the written informed consent, a finger prick blood sample was collected for haemoglobin (Hb) measurement using a handheld machine (Hemocue®) and G6PD screening by the fluorescent spot test (SQMMR500, R&D Diagnostics). If eligible, participants were referred to the trial clinician for randomisation and enrolment.

#### Enrolment, treatment and follow up

After successful screening, consent (day 0) and clinical review, each participant was randomized into one of the four study groups and followed up on days 1, 2, 3, 7, 10, 14, 21, 28, 35, and 42 or on any other day outside scheduled visits if they felt sick. At each visit, a blood sample (about 0.5ml by finger prick) was collected for the determination of gametocytaemia (QT-NASBA), parasite clearance (blood film and PCR) and Hb. In addition, a venous blood sample (3ml) was collected from a subset of 100 participants that consented for a direct membrane feeding assay to determine infectiousness to mosquitoes on day 7 (for more details see [25].

### Qualitative data collection

#### Participant observation

Participant observation is a key method in ethnographic research [31]. It involves participating in everyday activities in the community setting, observing events in their usual context and carrying out reiterated informal conversations with a maximum variety of research subjects. This method provides a more contextualised understanding of the research questions and helps cross-check the validity of the information obtained in semi-structured interviews. Participant observation reduces biased responses in interviews. Establishing a rapport and certain level of trust with the research participants is central to participant observation in ethnographic fieldwork. For this research, the main author and fieldworkers repeatedly visited and stayed in the village for a few days at a time over a time period of one year. During these stays, people’s everyday routines were observed, trust was established and informal conversations about relevant themes (i.e. the trial, the MRCG, blood, health and malaria) were held. Informal conversations were conducted in English and Fula and written down after the interview as soon as appropriate.

#### In-depth interviews

In-depth interviews were conducted at participants’ residences in private or in places where they felt most at ease. In total 30 interviews were conducted (Table 1). All formal in-depth interviews were recorded at this site and translated with the assistance of trained field assistants. These interviews were fully transcribed. The interviewees were visited and informally interviewed again on various occasions after the formal recording.

#### Group discussions

Two group discussions were conducted. The first (i) comprised a group of women who were looking after children in the same compound; this group discussion was about trial participation, the MRCG and blood-taking. The second (ii) was a group of men resting together at the *bantaba* (central meeting place). This discussion was about the history of the village, social structure and politics. The participation of various members enriched the data by providing different points of view in a discussion.

### Sampling

Participant recruitment was carried out through a mixed-sampling approach, including both purposive and snowball sampling techniques. Informants were purposively selected based on the questions emerging from the on-going analysis of collected data. Respondents were selected to ensure that all social groups were represented (maximum variation), regardless of local hierarchy or locally perceived expertise. In addition, snowball sampling was used to enhance participants’ trust and confidence in the research team when information was needed on sensitive subjects. Information about the respondents was collected and categorized in relation to relevant criteria, such as gender, age, social standing, trial participation and refusal, and role within the village [32].

### Data analysis

Data analysis was concurrent to data collection using a constructivist grounded theory approach [32]. Key analytic concepts in ethnographic research such as reflexivity, positionality and subjectivity were considered during the ethnographic fieldwork as well as during the data analysis. Relevant categories for analysis were established during the field research based on the participant observation, interviews and group discussions. Once patterns and themes relevant to the research question emerged, the results were constantly tested for their validity by including and confirming or refuting critical cases. The verification and validation of the results took place by systematically interviewing people belonging to different socio-demographic categories on the same topic, e.g. men versus women; trial participants versus non-participants; high versus low status groups. Once data saturation was reached for recurrent themes during the fieldwork, the results were further analysed and categorised after the transcription process. For the coding and analysis, the principle of retroduction was used combining inductive analysis from field data and theory from existing anthropological literature[24]. An initial coding framework was set up and further deepened through open coding. The analyses were carried out by means of a careful reading of the coded text fragments and with attention to potential interrelations. Through the analysing process data were constantly subjected to theoretical perspectives as to ensure theoretical triangulation and to embed the findings in existing literature. Data were imported, managed and analysed using NVivo 12 Qualitative Data Analysis software (QSR International Pty Ltd. Cardigan UK).

### Ethical considerations

The social science study was approved by the Gambia Government/MRC joint Ethics Committee (SCC number 1351) and the Institutional Review Board of the Institute of Tropical Medicine, Antwerp, Belgium (880/13). The interviewers followed the Code of Ethics of the American Anthropological Association (AAA). All interviewees were informed of the study, the type of questions to be asked and the intended use of the results prior to the interview. Anonymity and confidentiality were guaranteed and they were informed that they had the right to refuse or stop the interview any time. Verbal instead of written consent was preferred as requesting the subject’s signature could have been a potential reason for mistrust. A witness acknowledged the verbal consent and signed a documentation of consent sheet.

For ethical reasons and in order to avoid potentially negative repercussions for village inhabitants in the future (e.g. stigma), the name of the village and further details on the study subjects have been omitted from the manuscript.

## Results

### Details of screening and refusals

Among the 411 inhabitants of the village, 174 were approached and pre-screened for the trial. 43 people were absent or had moved away from the study area; 39 were not eligible because they were pregnant, breast-feeding or too old; 3 individuals refused to be screened and 149 were not pre-screened because the compound heads refused screening for the entire compound (5 out of 12 compounds) (Table 2).

**Table 2:** Details on pre-screening of Fula case study villages within the *Prinogam* study.

	n	%
**Status of pre-screening (N = 411)**		
Screened	174	42.3
Not screened	237	57.7
**Reason not screened (N = 237)**		
Travelled (absent)	40	16.9
Moved outside study area	3	1.3
Exclusion criteria:	42	17.7
*- Breast feeding*	*30*	*12*.*6*
*- Pregnant*	*4*	*1*.*7*
*- Too old (not being able to walk or give consent)*	*5*	*2*.*1*
*- Other reasons*	*3*	*1*.*3*
Individual refused	3	1.3
Whole compound refused	149	62.8

Although initially recruitment went smoothly, after one month the *Prinogam* study team encountered problems when no one wanted to be screened and some people refused to go to the hospital for follow-up.

### Reasons for refusal of blood sampling

#### Conceptions of blood as reasons for non-participation

The main reason for refusing participation was “blood taking”. Some trial participants spread the news that a lot of blood was taken at the hospital, which instigated rumours in the village leading to a sudden halt in screening due to the villagers’ complete refusal to participate. The given reason was fear of loss of strength through loss of blood, the latter associated with depleting life-force and body strength. Although people were told and had understood that the blood was taken to see if the person had malaria or not, most respondents believed that loss of blood was worse for a person’s health than the presence of malaria in the body. It was thought that the blood taken could not easily be replenished.

#### Blood as a life-force

Blood was inextricably linked to being alive and to health. Sickness was associated with lack of blood in the body, as illustrated in the following quote:

“The importance of blood is infinite. Blood is very, very important. Even for us here, who are sitting here talking, we are only able to do so because we are in good health. We are with the health that God lent us. No one can over-emphasize the importance of blood. When a bit of blood is missing from your body, you cannot be anything. It will affect some people’s brain, there is nothing negative that has not been said about blood taking.” *(Elderly herder*)

“For me when you talk about blood I think straight away about life because without blood, life will be very difficult to achieve. If you fall sick you might need blood because I think it circulates all over the body.” *(Middle aged herder*)

In respondents’ discourses, blood and work were closely linked. A person was thought not to be able to achieve much in life if the quality of their blood was not good. If a person was not able to endure long working hours on the fields or in the bush it was thought to be linked to their blood.

#### Nutrition and blood

Different conceptions of the ‘quality’ and ‘quantity’ of blood existed, which were linked to nutrition. Respondents reiterated that although they had ‘enough blood’ the ‘quality’ of their blood was not good because they were not eating well. People believed that their diet, which was almost entirely based on subsistence farming and herding, was not healthy. In order to replenish the strength lost through hard work, farming, cattle-herding or blood taking, more nourishing food was needed. The types of food that were thought to produce blood in the body were meat, oily food and rice.

“For us here, currently people are not strong because we are fully depending on cow milk. The groundnuts are just ripening. We don’t eat oil or meat; we only eat the fish people bring here for sale. We only eat our village food so where should we expect to get blood from? Even if you take our blood and put it in a small bottle it will cause us problems.” *(Elderly woman)*

“It’s because in the village here, the diet we take is in between bitterness and sourness and you know that cannot add blood in the body, our ways of surviving are not the same. Now if you are not surviving on a good diet and your blood isn’t much and people want to reduce that blood from you, it is going to be a problem. That is why most of the time, people in the village resist for their blood to be taken from them. If only you have enough money to eat good diets, diets that will give you vitamins, you will not be scared.” (*Elderly herder)*

“Before giving us food to replace the blood taken from our body, why don’t you just leave the blood that is in our body? [laughing] We are farmers and we don’t have blood. But right now the groundnuts are ripening and after eating enough groundnuts, if you take our blood we will not feel it. (…) You know, Fulas don’t like eating too much meat because they cannot buy it. We fully and surely depend on milk.” *(Elderly woman)*

#### Blood as fuel

It became evident from respondents’ explanations that the ‘quantity’ of blood was crucial for strength, which was thought to be needed on a day-to-day level to undertake the work necessary for subsistence farming. The more blood a person was thought to have, the stronger and more enduring they were believed to be farming “under the hot sun” or herding animals in the bush. If a person worked hard under the sun for extended periods of time, their blood was thought “to burn like fuel”, making him/her more vulnerable to weakness and sickness:

“(…) When your blood is taken and you have a lot of work to do in the sun, your blood will burn very well and that will make you scared so that for a few days you will not work. You will be asked to rest.” *(Young herder*)

Interviewer: “What is so important about blood? Is it that when you lose blood you will lose strength or do you think people can use the blood for evil?”

Respondent: “It is the strength you will lose of course because you see, even a car, it requires both petrol and water, if there is no water in the car it will obviously not go. Just like the petrol, if there is no petrol in the car it won’t go either. Is it not like that?” *(Middle aged herder)*

#### Blood and gender

Women were generally perceived to be more vulnerable to weakness than men because of pregnancy, child-birth and menstruation. Blood-donation was thought to be impossible due to health risks to the mother and the unborn child. Pregnancy was believed to last for at least eight months, in some cases up to two years. Thus it was thought that blood-taking was very bad for pregnant women, particularly those who had young children.

“I don’t know whether they don’t have enough blood but a woman is always giving birth. A woman who gives birth to a lot of children, if she gives out her blood, you know, that will be a problem. I am not saying that this is the case if you are not giving birth or when you have stopped giving birth. A woman cannot just start giving birth and give out blood, that is the problem.” *(Elderly woman)*

#### Blood testing, blood donations and blood stealing

People did not conceptually distinguish between blood sampling for testing and blood donation. Although the trial procedure and eligibility criteria of the trial had been explained on numerous occasions, many people seemed to think that some of the blood taken during the trial would be donated to someone or potentially sold. Although many of those enrolled in the trial said that too little blood was taken for the MRCG to use for other purposes and that they had seen them “check the blood with a machine”, those who refused to participate stated they did not have enough “blood to give away”. The following elderly herder compares trial participation to an event in his life when he was asked to donate blood:

“They asked me to donate two litres of blood but I insisted and only gave them a litre because that was what my daughter needed. But today with the condition that I am in, I know perfectly well that I cannot donate anyone even a litre of blood, I don’t have that amount of blood in my body because I really do know myself. My body is very weak as I am now.” (*Elderly herder)*

These beliefs arose particularly because the MRCG was not recruiting the ‘sick’ with malaria but asymptomatic carriers who were perceived to be completely healthy. Some believed that the drugs given to ‘healthy people’ were to make up for blood loss.

“… they say that if you are taken to the hospital, they will take your blood and in return the only thing they give you back is medicine, and we are told that this medicine is to replace the blood that has been taken from your body and that’s all they give you. They also said that you people are only after healthy people, those whose blood is good and healthy are the people you are after. You are not after the sick, and they are saying all that.” (*Middle aged herder)*

People believed that medical institutions, such as the MRCG, as well as hospitals in general would take more blood if they could get away with it. For some, it seemed a question of weighing up the benefits of trial participation against the risk of having one’s blood taken, which would then probably be sold or donated to someone else or used for sorcery.

#### Perceived risks of blood-taking

In addition to the more abstract conceptions of health and sickness as being linked to the quality of the blood, some respondents expressed more concrete fears related to blood-taking, such as losing so much blood that they would have to be hospitalised due to lack of blood, which would then require them to buy someone else’s blood:

“People are only suspicious of one thing and that is the issue of blood. So if you are taken to the hospital, and you don’t have enough blood, if they want to take your blood then you will be scared. Because if I lack blood today and I am taken to the hospital it may cost me a million to get blood, or 1500 Dalasi, 1000 Dalasi, or 500 Dalasi. The issue of blood is very hard and difficult. So this is the reason why people are suspicious of their blood being taking, if you need more blood and the little you have is about to be taken, you will obviously be scared.” *(Middle aged herder)*

Symptoms of weakness that were perceived to be linked to loss of blood were dizziness and loss of consciousness:

“I have seen many people, when their blood is taken or reduced they will faint and everything will be black in the person’s eyes. That is the problem. But there are some people who, when their blood is high, will make a request for their blood to be reduced and nothing will happen to the person.” *(Elderly woman*)

The latter quotation demonstrates the common belief that high blood pressure can be remedied through blood taking. It is thought that those suffering from high blood pressure should have their blood reduced regularly and that blood taking does not affect them in the same way as those who “do not have a lot of blood”.

## Discussion

The refusal to participate in public health campaigns or clinical trials in both African and European settings is commonly interpreted as caused by a lack of, or misunderstanding of the purpose of the intervention. ‘Myths’ and ‘rumours’ are often treated as irrational residues and by-products of people’s insufficient knowledge about new technologies and practices [33]. Additional documented factors leading to rumours are the lack of trust in public health institutions [33–35] and stigma related to trial participation [20]. This case study’s results show that concerns about daily subsistence and vulnerability related to poverty can also contribute to reticence. Those who refused were afraid of losing life-force and strength, which they associated with the quality of their blood. Particular concerns regarded the necessity to increase the quantity of blood in their body to restore strength. Previous research in the study region found that people evaluated different kinds of food in terms of their impact on blood and some foods are particularly sought after because they are thought to replenish blood and strength [36,37]. Those who refused to participate believed that the research team was going to give the blood to others and that they would be left weak, bloodless and vulnerable to sickness. Such beliefs seemed to be linked to the fact that only apparently ‘healthy’ people were being recruited in the trial and not those who were sick. Refusers felt that they were not in a position to “give away” their blood because they did not have enough and were afraid of getting sick if they lost more strength and, as a consequence, might need to get hospitalized and purchase blood for themselves. These findings are in line with Fairhead and Leach who describe that many people believe that blood taken during trials is later sold [11,36]. Cham (2003) described how, more than 12 years ago, patients in need of blood in The Gambia were given different options: either to receive blood from a close relative with the same blood group, or to buy blood for cash [38]. Such accounts may partially explain current blood-selling perceptions.

Vulnerability is a term commonly used to describe the dialectical relationship between poverty, risk and efforts to manage risks [39–42]. Locally perceived risk is socially constructed and depends not only on the nature of a hazard but also on political, social and cultural contexts [43,44]. Rather than solely being due to ignorance and misunderstanding, reluctance to participate may instead be linked to distinct forms of experiential expertise grounded in everyday practice, knowledge and epistemology [11,45]. It has been shown that in different socio-cultural contexts people feel vulnerable to different health problems, which do not always correspond to the calculated probability of epidemiological risk [46]. ‘Vulnerability refers to the actual feeling of susceptibility to illness or misfortune. It is a state of weakness, fear and worry’ [47]. In everyday language, risk is an expression of hazard, chance and uncertainty. In developed as well as in developing countries people think about and engage with risk and vulnerability in different ways. Whereas some members of an epidemiological risk group may not perceive themselves as vulnerable at all, for others exposure to risk data may trigger a sense of vulnerability [47]. As Gikonyo et al. [47] argued, locally perceived risks are often far greater and more dramatic than the biomedical risk mentioned in the informed consent procedures. In this study, the actual risk of taking a finger-prick of blood were minimal/non-existent but it was nevertheless perceived as potentially dangerous in the study village, clearly associated to perceptions of risk linked to poverty, lack of resources and possibly fear of exploitation. The fears around risk and threat to health, point to a social reality that needs to be addressed in informed consent procedures, public health research and policy planning.

It is not clear why these concerns reached the tipping point of non-participation in this study village, while there were few concerns about blood taking and trial participation in other *Prinogam* trial villages and research shows that people are often very keen to participate in clinical trials conducted by the MRCG in the Gambia [9]. The concrete results of this case study can therefore not be directly generalized to other trial villages in the Gambia. Nevertheless, individual reticence to trial participation in other places may also be linked to fear of blood taking due to depletion of life-force and fear of exploitation. Rumours can affect overall participation rates in research and disease elimination strategies. Further research might focus on how perceived risks can be appropriately addressed and discussed, for instance during the informed consent procedures.
